# Iatrogenic Kaposi’s Sarcoma: A Unique Case Unraveling Gastrointestinal Manifestations and Therapeutic Implications

**DOI:** 10.7759/cureus.57279

**Published:** 2024-03-30

**Authors:** Fatima Zahra Belabbes, Hajar Fadili, Abir Allaoui, Wafaa Kaikani, Fatima Zahra Agharbi

**Affiliations:** 1 Gastroenterology and Hepatology, Faculty of Medicine, Mohammed VI University of Health Sciences (UM6SS), Casablanca, MAR; 2 Laboratory Medicine, Faculty of Medicine, Mohammed VI University of Health Sciences (UM6SS), Casablanca, MAR; 3 Internal Medicine, Faculty of Medicine, Mohammed VI University of Health Sciences (UM6SS), Casablanca, MAR; 4 Medical Oncology, Faculty of Medicine, Mohammed VI University of Health Sciences (UM6SS), Casablanca, MAR; 5 Dermatology, Faculty of Medicine, Mohammed VI University of Health Sciences (UM6SS), Casablanca, MAR

**Keywords:** human herpesvirus 8, kaposi sarcoma paclitaxel, iatrogenic kaposi sarcoma, kaposi sarcoma hiv negative, dermatology, skin lesions, immunosuppression, gastrointestinal, kaposi, iatrogenic

## Abstract

Kaposi's sarcoma (KS), linked to human herpesvirus 8 (HHV8), manifests in various clinical forms with iatrogenic KS uniquely tied to immune dysregulation induced by medical interventions. This study describes a 58-year-old male of sub-Saharan origin with a medical history of segmental and focal hyalinosis treated with methylprednisolone and mycophenolate mofetil. The patient developed skin lesions on both thighs, accompanied by post-prandial vomiting and abdominal pain. Clinical examination revealed flesh-colored nodules on the thighs and inguinal lymphadenopathy. Biopsy confirmed the diagnosis of KS, exhibiting positive nuclear labeling to anti-HHV8 and negative HIV serology. Additionally, radiological findings from the thoracic-abdominal-pelvic computed tomography (CT) scan significantly contribute to our understanding of the multiorgan involvement associated with KS in this case, providing valuable insights for diagnosis and therapeutic considerations. This case highlights the iatrogenic subtype of KS, linked to immunosuppression from prior medical interventions. Notably, gastrointestinal involvement was evident, with lesions in the stomach and small intestine. Intravenous paclitaxel administration resulted in a positive clinical response. This study underscores the importance of clinical vigilance, endoscopic evaluation, and early intervention in the nuanced diagnosis and management of iatrogenic KS.

## Introduction

Kaposi's sarcoma (KS), a neoplasm originating from the endothelium and intricately linked to human herpesvirus 8 (HHV8), stands out as a prominent tumor in individuals with human immunodeficiency virus (HIV), capable of manifesting at any stage of the infection. Notably, KS can also emerge in patients undergoing immunosuppressive therapy [1]. While its primary presentation typically involves cutaneous lesions, gastrointestinal involvement remains relatively infrequent [2]. Despite its rarity in the gastrointestinal tract, asymptomatic digestive lesions are more prevalent than clinically apparent cases, contributing to the under-diagnosis of digestive KS [3]. When digestive symptoms do occur, they often lack specificity, encompassing manifestations such as diarrhea, vomiting, abdominal pain, and dyspepsia. Consequently, diagnostic endoscopy has emerged as a pivotal tool for uncovering latent digestive lesions in specific patients, including those with immunodeficiency disorders or undergoing chronic immunosuppressive therapy [4]. Recognizing the challenges posed by the nonspecific nature of symptoms and the often asymptomatic nature of digestive lesions, it is imperative to underscore the critical role of diagnostic procedures. Recent advances in chemotherapy offer a promising outlook, demonstrating positive outcomes in alleviating the morbidity and mortality associated with KS treatment.

This study endeavors to present a comprehensive overview of a unique case of KS, delving into clinical, histological, and radiological aspects. Particularly, the radiological findings from the thoracic-abdominal-pelvic computed tomography (CT) scan play a crucial role in enhancing our understanding of the intricate multiorgan involvement associated with KS.

## Case presentation

We present a case of a 58-year-old male patient of Sub-Saharan origin with a complex medical history. Two years prior to the current presentation, the patient received a diagnosis of segmental and focal hyalinosis. The management of this condition involved a methylprednisolone bolus and the immunosuppressant mycophenolate mofetil, administered at a daily dosage of 750 mg per day for a period of six months.

One year later, the patient began experiencing symptoms associated with his current condition. Notably, he developed skin lesions on both thighs, marking the commencement of his clinical presentation. The progression of these symptoms was accompanied by post-prandial vomiting and abdominal pain. Although there was no fever, the patient reported asthenia and fatigue.

**Figure 1:**
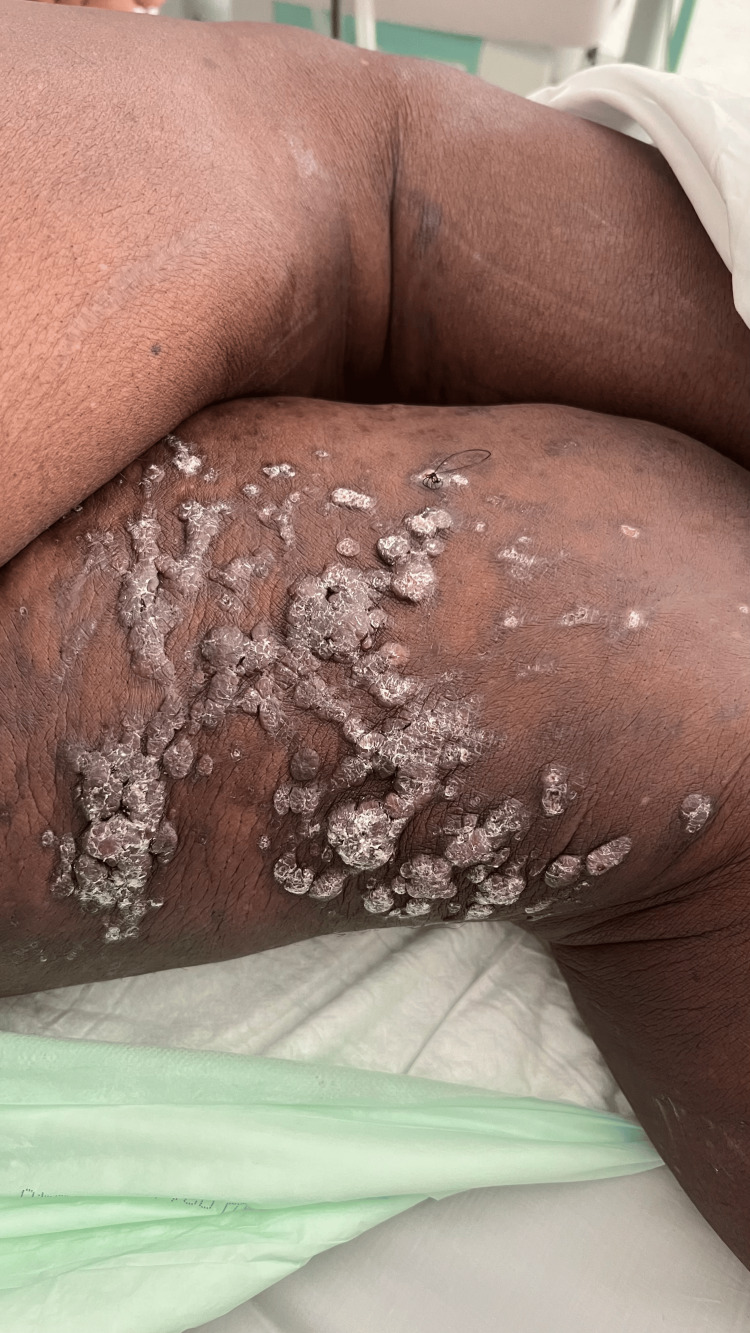
Dermatological manifestations of Kaposi's sarcoma. The lesions presented as centimeter-sized, violaceous erythematous nodules with warty surface, firm consistency, confluent in patches, located on both thighs.

**Figure 2:**
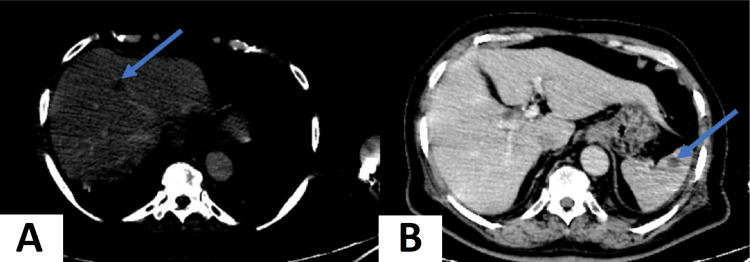
The thoracic-abdominal-pelvic computed tomography (CT) scan reveals well-defined rounded splenic and hepatic nodules without enhancement. (A) Axial section in the injected abdominal CT scan displays a rounded, well-limited, and not enhanced hepatic nodule in segment IV (arrow). (B) Axial slice in the injected abdominal CT scan depicts well-defined, rounded splenic nodules without enhancement (arrow).

The clinical findings include multiple flesh-colored nodules with a firm consistency identified on both thighs. This characteristic skin involvement prompted a closer inspection, revealing a diffuse distribution of these lesions (Figure 1). In addition, inguinal lymphadenopathy was noted during palpation. These lymph nodes appeared fixed and induced pain upon examination, suggesting a potential systemic involvement of the disease. The enhanced thoracic-abdominal-pelvic computed tomography (CT) scan disclosed well-defined, rounded splenic and hepatic nodules without enhancement (Figures 2A, 2B). 

**Figure 3:**
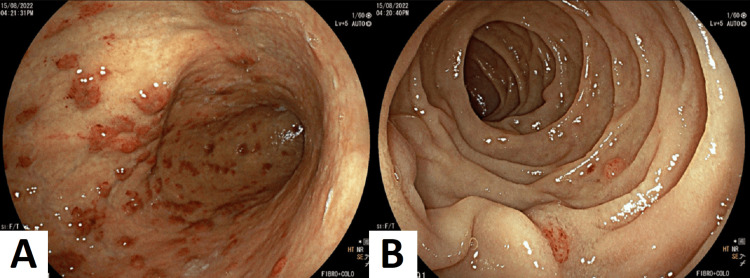
Esophagogastroduodenoscopy shows centimeter-sized, round reddish lesions with a slightly purplish hue. (A) The gastric body is displaying several centimeter-sized nummular reddish lesions, slightly purplish, and infiltrative. (B) The duodenal mucosa is displaying several centimeter-sized nummular reddish lesions, slightly purplish, and infiltrative.

**Figure 4:**
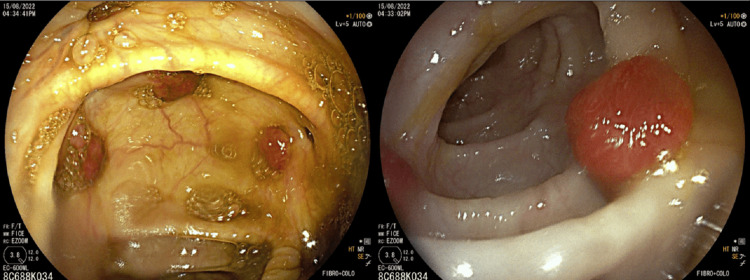
Colonoscopy shows scattered polypoid reddish centimeter-sized elevated round lesions, diffusely distributed over the entire colonic mucosa.

Esophagogastroduodenoscopy revealed centimeter-sized, round reddish lesions with a slightly purplish hue. These infiltrative lesions were diffusely distributed throughout the gastric body and duodenal mucosa (Figures 3A, 3B). Conversely, colonoscopy revealed scattered polypoid reddish lesions. These elevated round lesions, measuring several centimeters in size, were diffusely present across the entire colonic and omental mucosa (Figure 4).

Following the clinical examination, a biopsy of the skin lesions was conducted, confirming the diagnosis of KS. A pathological study of the gastrointestinal lesions definitively established the diagnosis of KS, with positive nuclear labeling observed in response to anti-HHV8 staining. Notably, HIV serology returned negative. Following the diagnosis, the patient underwent a therapeutic intervention, receiving intravenous paclitaxel at a dosage of 100 mg weekly over a span of nine weeks. Subsequent to the initiation of this treatment regimen, a notable clinical improvement was observed.

## Discussion

KS, originating from the endothelium and associated with KSHV, typically manifests in conditions of immune dysregulation, as observed in the presented case of iatrogenic KS. This form is notably associated with a poorer prognosis compared to other KS types, because patients under immunosuppression for causes other than HIV, particularly those related to medication associated with autoimmune diseases, are more susceptible to HV-8 infection and its manifestation of KS. This implies comorbidity that worsens their prognosis, and its prevalence may increase due to the progressive rise in the diagnosis of rheumatological diseases, the early use of immunosuppressive therapies, as well as the increase in various organ transplants and the improved survival achieved by these patients [5].

Our patient, a 58-year-old male of sub-Saharan origin with a history of segmental and focal hyalinosis, was treated with a methylprednisolone bolus and mycophenolate mofetil for six months. The symptoms began one year later, with skin lesions on the thighs. This clinical presentation aligns with iatrogenic KS [6].

Gastrointestinal (GI) involvement in KS is of particular relevance to our case. While the GI tract can be affected from the oropharynx to the rectum, our patient exhibited lesions in the stomach and small intestine. Additionally, the skin lesions observed in the clinical examination parallel the classic manifestation of KS, primarily affecting the skin.

Despite being the third most common location for KS, gastrointestinal involvement carries significant clinical implications. Lesions may remain asymptomatic, contributing to underdiagnosis, or lead to symptoms such as abdominal pain, vomiting, and anemia, as observed in our patient [7]. In some cases, as with our patient, KS can cause acute gastrointestinal bleeding, a severe complication rarely reported in the literature [8]. This underscores the importance of recognizing potential gastrointestinal KS in patients with digestive symptoms, especially those with a history of immunosuppression.

In this case, the diagnostic endoscopy not only confirmed the presence of KS lesions in the stomach and small intestine but also provided the necessary histological and immunohistochemical confirmation, crucial for an accurate diagnosis. Understanding the endoscopic findings is vital; in our patient, it included red maculopapular lesions, darker nodular, or polypoid lesions, aligning with the spectrum of appearances associated with gastrointestinal KS.

The radiological findings uncovered through the thoracic-abdominal-pelvic computed tomography (CT) scan significantly contribute to our comprehension of the intricate multiorgan manifestations of KS in the presented case. This comprehensive insight not only aids in recognizing the extent of organ involvement but also provides essential information for guiding diagnostic and therapeutic strategies [9].

The unique aspects of our case, including iatrogenic KS, skin lesions, and gastrointestinal involvement, emphasize the importance of tailored diagnostic approaches and considerations for therapeutic interventions, such as the administration of intravenous paclitaxel, which led to a notable clinical improvement [10].

## Conclusions

In conclusion, our case report emphasizes the intricate link between iatrogenic KS and immunosuppression. Our findings contribute to understanding this condition, emphasizing the significance of vigilance in clinical, endoscopic assessments and histological confirmation for early detection. In navigating immunosuppression-related malignancies, this report underscores the need for nuanced diagnostic approaches and targeted treatments in managing iatrogenic KS. The positive response to intravenous paclitaxel suggests tailored therapeutic potential in iatrogenic KS, offering hope for improved outcomes.
